# Introduction and establishment of fluoroquinolone-resistant *Shigella sonnei* into Bhutan

**DOI:** 10.1099/mgen.0.000042

**Published:** 2015-12-24

**Authors:** Hao Chung The, Maia A. Rabaa, Duy Pham Thanh, Sirigade Ruekit, Sonam Wangchuk, Tshering Dorji, Kinzang Pem Tshering, To Nguyen Thi Nguyen, Phat Voong Vinh, Tuyen Ha Thanh, Chau Nguyen Ngoc Minh, Paul Turner, Poda Sar, Guy Thwaites, Kathryn E. Holt, Nicholas R. Thomson, Ladaporn Bodhidatta, Carl Jeffries Mason, Stephen Baker

**Affiliations:** ^1^​ The Hospital for Tropical Diseases, OUCRU, Ho Chi Minh City, Vietnam; ^2^​ Centre for Tropical Medicine, Oxford University, Oxford, UK; ^3^​ Department of Enteric Diseases, AFRIMS, Bangkok, Thailand; ^4^​ Public Health Laboratory, Department of Public Health, Ministry of Health, Royal Government of Bhutan, Thimphu, Bhutan; ^5^​ Department of Pediatrics, Jigme Dorji Wangchuk Referral Hospital, Kawa Jangsa, Thimphu, Bhutan; ^6^​ Cambodia–Oxford Medical Research Unit, Angkor Hospital for Children, Siem Reap, Cambodia; ^7^​ Department of Biochemistry and Molecular Biology, Bio21 Molecular Science and Biotechnology Institute, University of Melbourne, Parkville, Victoria 3010, Australia; ^8^​ The Wellcome Trust Sanger Institute, Hinxton, Cambridgeshire, UK; ^9^​ The London School of Hygiene and Tropical Medicine, London, UK

**Keywords:** Bhutan, fluoroquinolone, resistance, *Shigella sonnei*

## Abstract

*Shigella sonnei* is a major contributor to the global burden of diarrhoeal disease, generally associated with dysenteric diarrhoea in developed countries but also emerging in developing countries. The reason for the recent success of *S. sonnei* is unknown, but is likely catalysed by its ability to acquire resistance against multiple antimicrobials. Between 2011 and 2013, *S. sonnei* exhibiting resistance to fluoroquinolones, the first-line treatment recommended for shigellosis, emerged in Bhutan. Aiming to reconstruct the introduction and establishment of fluoroquinolone-resistant *S. sonnei* populations in Bhutan, we performed whole-genome sequencing on 71 *S. sonnei* samples isolated in Bhutan between 2011 and 2013.We found that these strains represented an expansion of a clade within the previously described lineage III, found specifically in Central Asia. Temporal phylogenetic reconstruction demonstrated that all of the sequenced Bhutanese *S. sonnei* diverged from a single ancestor that was introduced into Bhutan around 2006. Our data additionally predicted that fluoroquinolone resistance, conferred by mutations in *gyrA* and *parC*, arose prior to the introduction of the founder strain into Bhutan. Once established in Bhutan, these *S. sonnei* had access to a broad gene pool, as indicated by the acquisition of extended-spectrum β-lactamase-encoding plasmids and genes encoding type IV pili. The data presented here outline a model for the introduction and maintenance of fluoroquinolone-resistant *S. sonnei* in a new setting. Given the current circulation of fluoroquinolone-resistant *S. sonnei* in Asia, we speculate that this pattern of introduction is being recapitulated across the region and beyond.

## Data Summary

1. Whole-genome sequence reads of *Shigella sonnei* used in this study have been deposited to the European Nucleotide Archive. The run accession numbers and related metadata are detailed in Table S1 which has been deposited in FigShare: http://figshare.com/articles/The_introduction_and_establishment_of_fluoroquinolone_resistant_*Shigella*_sonnei_into_Bhutan/1610693

## Impact Statement

The bacterium *Shigella sonnei* is a major global cause of diarrhoeal disease. This organism has generally been associated with infections in developed countries, but is currently dramatically expanding into industrializing countries. The mechanisms underpinning the spread of *S. sonnei* are not precisely understood, but we hypothesize that their spread is partly facilitated by resistance to antimicrobials, including the current therapeutic choice to combat *Shigella*, the fluoroquinolones. To understand how the fluoroquinolone-resistant *S. sonnei* may become established in a new location, we decoded the total DNA sequences of 71 *S. sonnei* samples isolated in Bhutan in South Asia between 2011 and 2013. We found that these strains were likely descended from a single isolate that was introduced into Bhutan around 2006. Furthermore, we additionally found that fluoroquinolone resistance emerged prior to the introduction of this founder *S. sonnei* into Bhutan and these strains then became established, gaining additional genes and resistance to other antimicrobials. Our data describe how drug-resistant *S. sonnei* may become resident in new locations, and we predict that a similar situation is occurring currently with the epidemic of fluoroquinolone-resistant *S. sonnei* in Asia and beyond.

## Introduction

It has recently been estimated that the global burden of diarrhoeal disease in children < 5 years of age is ∼1.7 billion new infections with 0.7 million deaths per year (Walker *et al.*, 2013). These figures make diarrhoeal diseases, after respiratory tract infections, the second most common cause of mortality in young children in low-income countries (Liu *et al.*, 2012; WHO, 2014). Further contemporary data from the Global Enteric Multicentre Study, the largest prospective case–control study on paediatric diarrhoeal illnesses in sub-Saharan Africa and South Asia ever conducted, identified *Shigella* to be amongst the most common diarrhoeal pathogens in these high-incidence settings (Kotloff *et al.*, 2012, 2013).

The members of the genus *Shigella* are Gram-negative pathogens that are closely related to *Escherichia coli* (Pupo *et al.*, 2000) and cause dysenteric diarrhoea (stools containing blood and/or mucus) via a cascade of virulence factors encoded principally on a large signature virulence plasmid (Sansonetti *et al.*, 1982). The genus contains four species: *Shigella sonnei*, *Shigella flexneri*, *Shigella boydii* and *Shigella dysenteriae*, with *S. sonnei* and *S. flexneri* dominating in developed and developing countries, respectively. However, this pattern is changing, as *S. sonnei* is currently replacing *S. flexneri* in countries undergoing economic transition (Vinh *et al.*, 2009; Thompson *et al.*, 2015). This species replacement has positive and negative ramifications. By replacing *S*. *flexneri*, which is very diverse with multiple serotypes with respect to the single serotype *S. sonnei* (Connor *et al.*, 2015), as the leading cause of dysentery means that it may be possible to control disease through vaccination (Levine *et al.*, 2007). However, *S. sonnei* is also highly adept at acquiring antimicrobial resistance genes and resistance-associated mutations (Huang *et al.*, 2005; Seol *et al.*, 2006; Nguyen *et al.*, 2010), limiting treatment options and making control through antimicrobial treatment alone improbable.

Recent advances in whole-genome sequencing (WGS) and analytical methodologies have enhanced our understanding of the epidemiology, international spread and antimicrobial susceptibility trends of *S. sonnei* (Holt *et al.*, 2012, 2013). We now know that *S. sonnei* is formed of three main lineages (I, II and III), which are all related to a common ancestor that arose in Europe ∼400 years ago (Holt *et al.*, 2012). Lineage III, which is multidrug-resistant (MDR), now dominates globally (Holt *et al.*, 2012). We have also previously shown that once introduced into a new geographical region, into an apparently naive human population, *S. sonnei* can establish new isolated clonal populations which evolve locally (Holt *et al.*, 2013). Antimicrobial resistance is a key driver of the recent *S. sonnei* global expansions marked by the acquisition of antimicrobial resistance genes and/or mutations in genes encoding the target proteins for antimicrobials (Pu *et al.*, 2015).

Bhutan is a lower middle-income country in South Asia, sandwiched between China and the northwest Indian states of Assam and West Bengal. In close proximity to Nepal ( < 70 km of Indian territory separates the two countries), Bhutan is a Himalayan nation with an estimated population of 750 000 people, of which the majority (69 %) live in remote rural locations (Office of the Census Commissioner – Royal Government of Bhutan, 2006). Like other low-income countries in the region, diarrhoeal disease is a major public health problem in Bhutan, and both *S. flexneri* and *S. sonnei* are endemic (Marfin *et al.*, 1994; Ruekit *et al.*, 2014). Between March 2011 and October 2013, a diarrhoeal disease surveillance study was conducted in the Bhutanese capital, Thimphu. During this study, there was an increased number of *S. sonnei* isolated between June and July. Antimicrobial susceptibility testing, PFGE and plasmid replicon typing of 29 of these *S. sonnei* isolates showed that all were identical by PFGE pattern and antimicrobial susceptibility (Ruekit *et al.*, 2014). All these *S. sonnei* isolates were resistant to ciprofloxacin (MIC ≥ 3 μg μl^− 1^), which was infrequently observed in strains circulating globally during the same period (Ruekit *et al.*, 2014). This study suggested that these *S. sonnei* isolates were closely related and may have been a recent introduction into Bhutan.

To test the hypothesis that these fluoroquinolone-resistant *S. sonnei* had recently been introduced to Bhutan and to further characterize these Bhutanese isolates in the context of global *S. sonnei*, we performed WGS on 71 *S. sonnei* isolated in Bhutan between 2011 and 2013.

## Methods

### Sample collection

The Research Ethics Board of Health in Bhutan and the Institutional Review Board of the Walter Reed Army Institute of Research provided ethical approval for the study. Stool samples were collected as described previously (Ruekit *et al.*, 2014). Stool samples were collected from cases and controls, and cultured on MacConkey and Hektoen agar, and incubated overnight at 37 °C. Non-lactose-fermenting colonies were confirmed to be *Shigella* by standard biochemical testing; serotype was identified by O-antigen agglutination (Denka Seiken).

Stool samples were collected during a diarrhoea surveillance study conducted at the Jigme Dorji Wangchuk National Referral Hospital (JDWNRH) in Thimphu, Bhutan, between March 2011 and October 2013, as described previously (Ruekit *et al.*, 2014). Briefly, children aged between 3 months and 5 years presenting to JDWNRH with acute diarrhoea (three or more loose stools in the previous 24 h, with symptoms lasting ≤ 72 h at time of recruitment) and those with no diarrhoeal symptoms in the previous 2 weeks were eligible for recruitment as cases and controls, respectively (informed consent permitting).

All *Shigella* isolates were subjected to antimicrobial susceptibility testing using the disk diffusion method as recommended by Clinical and Laboratory Standards Institute guidelines (CLSI, 2012). The following antimicrobials were selected for susceptibility testing: azithromycin, nalidixic acid, ciprofloxacin, ampicillin, trimethoprim/sulfamethoxazole, ceftriaxone, streptomycin and tetracycline.

### WGS

During the surveillance study a total of 112 *Shigella* were isolated (74 *S. sonnei*, 30 *S. flexneri*, seven *S. boydii* and one *S. dysenteriae*); the 74 *S. sonnei* were selected for WGS. The majority (64/74) of the selected *S. sonnei* were isolated from symptomatic diarrhoeal stool samples with the remaining strains isolated from asymptomatic infections.

Genomic DNA was extracted from all *S. sonnei* isolates using a Wizard Genomic DNA Extraction kit (Promega) according to the manufacturer's recommendations. For each sample, 2 μg genomic DNA was subjected to WGS on an Illumina HiSeq2000 platform, following the manufacturer's protocols to generate 100 bp paired-end reads (Quail *et al.*, 2008). All reads were mapped to the reference sequence of *S. sonnei* Ss046 (GenBank accession number NC_007384) using smalt (version 0.7.4). Candidate SNPs were called against the reference sequence and filtered using SAMtools (Li *et al.*, 2009). Low-quality SNPs were removed according to the following criteria: consensus quality < 50, mapping quality < 30, ratio of SNPs to reads at a position < 75 %, read depth < 4, number of reads per strand < 2, strand bias < 0.001, mapping bias < 0.001 or tail bias < 0.001. Three samples failed due to insufficient read depth, resulting in a final sample set including 71 *S. sonnei* whole-genome sequences from Bhutan.

### Phylogenetic analysis, temporal analysis and Bayesian phylogenetic inference

In order to place the Bhutanese isolates in the context of global diversity, we aligned the Bhutanese *S. sonnei* sequences with publicly available sequences utilized in a previous global analysis (Holt *et al.*, 2012). An initial phylogeny was inferred with these data using RAxML under the GTRGAMMA model. To further assess the relationship of the Bhutanese isolates within Central Asia, the previous alignment was then subsampled to include only the 71 Bhutanese sequences along with 20 other closely related Central Asia sequences. Previously described mobile genetic elements (Holt *et al.*, 2012) and potential recombinant regions were removed from the alignment using Gubbins (Croucher *et al.*, 2014). Further removal of gaps, indeterminate bases, invariant sites and 17 Bhutanese sequences that were identical to others in the dataset resulted in a final alignment of 996 SNPs across 74 taxa. A maximum-likelihood phylogeny was inferred from these sequences using garli (Zwickl, 2006) with 1000 bootstrap replicates under the TVM (transversion) model of nucleotide substitution, which was determined to be the best-fit model using jModelTest (Posada, 2008).

In order to thoroughly examine the population and temporal structure of Bhutanese *S. sonnei* and the population from which they emerged, all sequences from Bhutan, including identical sequences removed in the previous phylogeny, were aligned with the six most closely related isolates from other countries and trimmed down to include only the 544 SNPs present in the alignment of 77 taxa. These data were then utilized for maximum-likelihood reconstruction using PhyML under the TVM+Γ_4_ substitution model (Guindon *et al.*, 2010). In order to investigate whether temporal signal was embedded in the resulting maximum-likelihood phylogeny, we applied Path-O-Gen (version 1.4) to assess the linear relationship between root-to-tip divergence and date of isolation (in day, month, year).

Bayesian MCMC (Markov Chain Monte Carlo) deployed in beast (version 1.8.0) (Drummond & Rambaut, 2007) was then used to infer the evolutionary dynamics of the aforementioned dataset, with a particular focus on Bhutanese *S. sonnei*. To determine the best-fit priors to the data at hand, multiple beast runs were conducted using the TVM or GTR nucleotide substitution model with constant, exponential growth or Bayesian skyline demographic history priors, in combination with either a strict or a relaxed molecular clock (uncorrelated lognormal distribution) (Drummond & Rambaut, 2007). For each beast analysis, marginal likelihood estimation was conducted using both path sampling and stepping-stone sampling approaches to facilitate model selection (Baele *et al.*, 2012, 2013). Bayes factor comparison indicated the TVM+Γ_4_ substitution model with a relaxed log-normal molecular clock and a piecewise Bayesian skyline demographic model as the best-fit model to the data. Analyses using these priors were then performed in triplicate using a continuous 100 million generation MCMC chain with samples taken every 10 000 generations and parameter convergence (indicated by effective sample size values >200) was assessed in Tracer (version 1.5). LogCombiner (version 1.8) was used to combine triplicate runs, with removal of 20 % burn-in.

### Gene content analysis

*De novo* assembly for each read set was performed using the assembler Velvet (version 1.2.03) and VelvetOptimizer, with each read set mapped back to each assembly (Zerbino & Birney, 2008). Contigs < 300 bp were discarded. Assemblies were annotated using the rapid prokaryotic genome annotation tool Prokka (Seemann, 2014). To identify contigs associated with the accessory genome, contigs were first-ordered with *S. sonnei* Ss046 and its large virulence plasmid pSS046 as references using abacas. The remaining contigs were visualized and examined in Artemis. Presence of specific accessory genetic determinants was confirmed by blast and visualized using act (Carver *et al.*, 2008).

For each isolate, the entire resistance gene content (resistome) was identified by mapping the assembly to a manually curated resistome database as described previously (Chung The *et al.*, 2015). MLST was performed for all *Shigella* isolates by profiling the seven MLST genes (*adk*, *fumC*, *gyrB*, *icd*, *mdh*, *purA*, *recA*). Mutations in specific genes were manually assessed by retrieving their alignments from the annotations and the effects of non-synonymous mutations were predicted using the sift web server (http://sift.jcvi.org/) (Sim *et al.*, 2012). An *in silico* plasmid incompatibility-type PCR was performed using an in-house script and bespoke primer set.

## Results

### Bhutanese *S. sonnei* lies within lineage III (Central Asia clade)

**Fig. 1.**
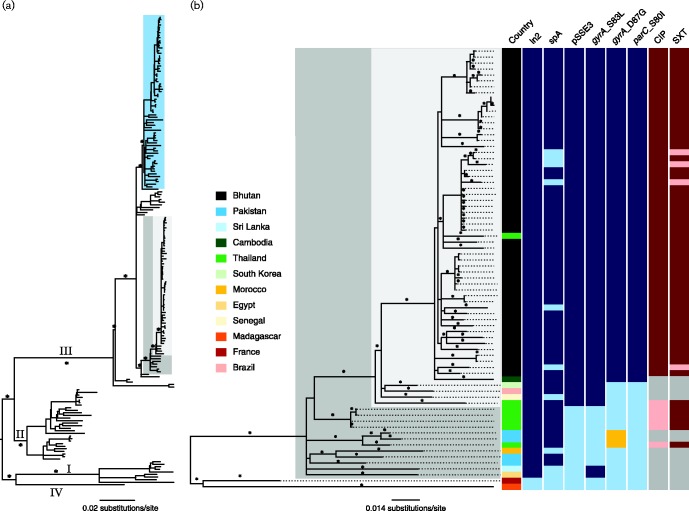
The phylogenetic structure of Bhutanese *S. sonnei* in the context of the global phylogeny and Central Asia clade. (A) Midpoint rooted maximum-likelihood phylogenetic tree of 183 *S. sonnei* strains (135 from global collection and 48 from Bhutan) reconstructed using 5393 SNPs; asterisks indicate bootstrap support values ≥ 98 % on major branches. Numbers above major branches represent ones leading to major lineages (I, II, III and IV). The light blue box highlights the Global III clade; the dark grey box overlaid on the tree highlights strains belonging to the Central Asia clade; the smaller, light grey box highlights the primarily Bhutanese Central Asia clade. (B) Magnified view of the maximum-likelihood phylogenetic tree of the Central Asia clade, including 74 *S. sonnei* strains (54 from Bhutan and 20 others for phylogenetic context), reconstructed using 996 SNPs; asterisks indicate bootstrap support values ≥ 80 %. The tree is midpoint rooted for purposes of clarity. Columns to the right of the phylogenetic tree show isolate metadata including: country of isolation (colours indicated in the key); the presence (dark blue) or absence (light blue) of specific genetic elements and mutations [ln2, spA, pSSE3, *gyrA* (S83L), *gyrA* (D87G), *parC*(S80I)], or the presence of an alternative mutation (yellow) [*gyrA* (D87Y)]; and resistance profiles against ciprofloxacin (CIP) and trimethoprim/sulfamethoxazole (SXT), where resistance is indicated in dark red, susceptibility in light red and missing data in grey.

All *S. sonnei* (*n* = 74) isolated during this study were subjected to WGS; 71 isolates produced sufficient sequence for analysis. To contextualize the Bhutanese *S. sonnei* within the known global *S. sonnei* population structure, we constructed a phylogeny that included the Bhutanese sequences and those from a previous global *S. sonnei* sequencing project (Holt *et al.*, 2012); within this phylogeny, all Bhutanese *S. sonnei* fell into a single clade within lineage III (Central Asia clade), which is a sister clade to the intercontinental expanding Global III clade (Fig. 1A). To investigate the phylogenetic relationships within the Central Asia clade, we concatenated 74 of the non-duplicate whole-genome sequences, including 54 from Bhutan and 20 selected from other countries. A maximum-likelihood phylogeny reconstructed from this alignment showed a close clonal relationship between the Bhutanese *S. sonnei*. Showing a similarly close relationship to the Bhutanese isolates, and interspersed within this clade, were also two isolates from Thailand and Cambodia, likely representing importations following regional travel (Fig. 1B).

### Temporal phylogenetic reconstruction of *S. sonnei* in Bhutan

**Fig. 2.**
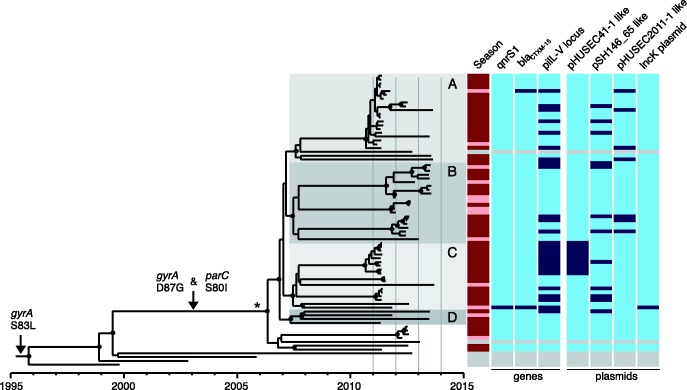
Temporal phylogenetic reconstruction of Bhutanese *Shigella sonnei* between 2011 and 2013.Image shows a maximum clade credibility phylogenetic reconstruction of *S. sonnei* isolated primarily in Bhutan over the study period. Distinct subpopulations of Bhutanese *S. sonnei* are highlighted and indicated by letters. Black circles indicate posterior probability support ≥ 80 % on internal nodes. The asterisk indicates the branch leading to the Bhutanese *S. sonnei* clade, which represents 22 lineage-defining SNPs. Arrows denote branches characterized by select substitution events. Columns to the right of the phylogeny show metadata including: season (dark red, monsoon season: June–September; light red, other), and presence (dark blue) or absence (light blue) of mobile genetic elements, including specific genes (*qnrS1*, *bla*_CTXM-15_, *pilL–V* locus) and plasmids (pHUSEC41-1-like, pSH146_65-like, pHUSEC2011-1-like, IncK). Grey bars in metadata columns indicate sequences from *S. sonnei* isolated outside of Bhutan.

As there was such a strong temporal signature for the number of observed mutations in the global *S. sonnei* population (Holt *et al.*, 2013), we explored the temporal structure of the *S. sonnei* isolated in Bhutan using Bayesian phylogenetic methods. To do this, the nucleotide substitution rate of the primarily Bhutanese lineage of Central Asia was determined to be 7.34 × 10^− 7^ substitutions per site per year [95 % highest posterior density (HPD): 5.87 × 10^− 7^ to 8.83 × 10^− 7^], which is comparable to the previously calculated substitution rate of *S. sonnei* lineage III (Holt *et al.*, 2013). Using these data, we estimate that the most recent common ancestor (MRCA) of the Bhutanese *S. sonnei* existed around 2006 (95 % HPD 2005–2007), with the majority diverging from a hypothetical MRCA around 2007 (95 % HPD 2005–2008) (Fig. 2). The results of this analysis were further supported by the linear relationship between root-to-tip branch length and date of isolation (*R*^2^ = 0.8412). Since its projected introduction in 2006, this *S. sonnei* lineage diverged into at least four sublineages (labelled A, B, C and D in Fig. 2), which have been transmitted endemically within the Bhutanese capital, Thimphu: isolates taken from cases in June/July 2011 (25/71 isolates) were found to belong to two subpopulations (A and C). Sublineage B, which was not represented in June/July 2011, dominated in the subsequent years (Fig. 2).

### Mutations specific to Bhutanese *S. sonnei*

To identify potential genetic mechanisms associated with diversification of the Bhutanese *S. sonnei*, we compared their content of unique SNPs in comparison with the three most closely related outgroup isolates, taken from global isolates belonging to the same global lineage but collected outside of Bhutan (Fig. 2). The Bhutanese isolates were distinguished from these by the presence of 22 lineage-specific consensus SNPs. Of these 22 SNPs, five fell in intergenic regions and 17 mapped within predicted coding sequences; 11 represented non-synonymous and six were synonymous mutations. The genes bearing non-synonymous mutations are summarized in Table 1 together with their predicted impact on function. These include mutations in *gyrA* and *parC* associated with antimicrobial resistance, as well as those located in *ycgB*, *mppA*, *hisC* and *rbsK*, as determined by *in silico* sift (see Methods) analysis. With the exception of *ycgB* encoding a protein with unknown function, these genes all encode proteins predicted to be involved in the metabolism of histidine, ribose and formate, as well as transport of murein tripeptide (Table 1).

**Table 1.** Non-synonymous mutations separating the MRCA of all Bhutanese *S. sonnei* from other Central Asia clade strains

Gene	Mutation	Function	sift score*/effect
*cusS*	G343S	Sensory histidine kinase of CusSR, regulating the expression of the CusCFBA operon to confer resistance to high copper and silver concentrations	0.77
*ycgB*	Y125H	Unknown function	0
*mppA*	G344R	Periplasmic binding component of murein tripeptide in oligopeptide transport	0
*hisC*	P98S	Histidinol-phosphate aminotransferase, catalysing the biosynthesis of histidine	0.03
*eutL*	A22S	Carboxysomal shell protein, structural component of proteinaceous microcompartment for ethanolamine ammonia lyase	0.5
*hycC*	G538D	Component of HycBCDEFG, hydrogenase component (hydrogenase 3) of formate hydrogenlyase; HycC encodes an extremely hydrophobic protein with homology to NADH : ubiquinone oxidoreductase	0
*sdaB*	A407S	l-Serine deaminase II, catalysing the conversion of serine into pyruvate and ammonia	0.33
*metC*	A2T	Cystathionine B-lyase and cysteine desulfhydrase, catalysing the conversion cystathionine to homocysteine	0.7
*rbsK*	A50T	Ribokinase responsible for metabolism of d-ribose	0
*gyrA*	D87G	DNA gyrase, subunit A	*Known effect*: resistance to fluoroquinolones
*parC*	S80I	DNA topoisomerase IV subunit A	*Known effect*: resistance to fluoroquinolones

* sift scores ≤ 0.05 indicate a significant effect on protein function based on sift analysis.

### Accessory genome and impact of antimicrobial resistance genes in Bhutanese *S. sonnei*

The composition of the accessory genome can be used to indicate the capacity of the host species to sample the local gene pool and also to highlight themes in the patterns of acquisition that may be under selection. We performed a comparative gene content analysis of Bhutanese and non-Bhutanese *S. sonnei* belonging to the Central Asia clade. These data revealed events that appear to have been pivotal in evolution and spread of the Central Asia clade. Notably, all the isolates of this clade possessed a specific class 2 integron In2 (*dfrA1*, *sat1*), conferring resistance to trimethoprim and streptothricin. These strains additionally gained the *S. sonnei* spA plasmid carrying *sul2*, *strAB* and *tetRA* genes, which further expanded the spectrum of antimicrobial resistance to incorporate sulphonamides, streptomycin and tetracycline resistance, respectively (Fig. 1B). This resistance gene profile concurs with the observation made in the previous global *S. sonnei* study (Holt *et al.*, 2012). It is of note that the class 2 integron of the Central Asia clade differs from that of Global III in the absence of *aadA1*, which confers resistance to aminoglycosides. Moreover, it was clear that isolates within this clade have, over time, accumulated chromosomal mutations conferring fluoroquinolone resistance (Fig. 1B). From the lineage-specific phylogeny, we predicted that the first mutation in *gyrA* (S83L) occurred before 1996, and supplementary mutations in *parC* (S80I) and secondary mutations in *gyrA* (D87G), leading to high-level fluoroquinolone resistance, were fixed in the Bhutanese clade prior to its expansion and diversification in 2006 (Fig. 2).

**Fig. 3.**
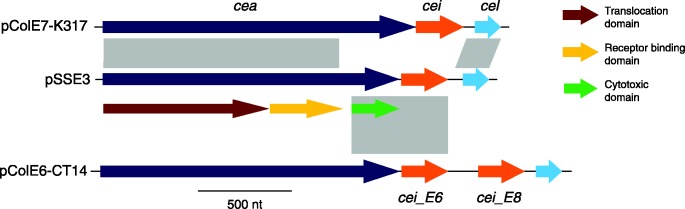
Novel recombinant hybrid colicin biosynthesis cluster encoded on pSSE3.Schematic representation of the colicin biosynthesis cluster on plasmid pSSE3 in comparison with colicin gene clusters of pColE7-K317 and pColE6-CT14. Grey vertical bars show homology in DNA sequences (with identity >80 %). The colicin structural gene (*cea*), immunity gene (*cei*) and lysis gene (*cel*) are annotated as shown. The three domains of the colicin structural gene are shown the key.

A further pivotal event in the emergence of this Central Asia clade prior to its introduction into Bhutan was the fixation of the previously described E-type colicin plasmid pSSE3 (with 100 % identity in DNA sequence). This E-type colicin has been shown to be antagonistic against a variety of diarrhoeogenic *E. coli*, but not against commensal *E. coli* (Calcuttawala *et al.*, 2015). Plasmid pSSE3, which was found in all Bhutanese isolates (Fig. 1B), also carries an immunity function that protects the host cell against the activity of the corresponding colicin. Although it was previously characterized as a ColE3 plasmid by PCR, sequence analysis of pSSE3 indicated that the colicin biosynthesis cluster was a novel recombinant hybrid with an undetermined spectrum of activity (Fig. 3). On closer examination, the first and second domains (translocation and receptor-binding) of the colicin structural gene (*cea*) and the lysis gene (*cel*) exhibited substantial amino acid sequence identity (98 %) to that of colicin E7 (ColE7). The third domain (cytotoxic) encoded by *cea* was most closely related to that of ColE6 (94 % protein sequence identity), predicting that the colicin's target is actually 16S rRNA rather than DNA, which is the target of the ColE7 counterpart (Kleanthous, 2010). However, the product of the colicin immunity gene (*cei*) exhibited only 84 % protein sequence identity to that of colicin E6.

Genetic material that was acquired sporadically across the Central Asia clade within the Bhutanese isolates included the extended-spectrum β-lactamase (ESBL) gene *bla*_CTXM-15_, which was independently acquired by two *S. sonnei* on different incompatibility plasmids (IncK and IncI1), conferring resistance to third-generation cephalosporins (Fig. 2). The plasmid replication and transfer region of the IncK plasmid shared substantial sequence homology with that of the enteroaggregative *E. coli* IncI1 plasmid pSERB1. This plasmid in the Bhutanese *S. sonnei* incorporated the type IV pilus biosynthesis cluster *pilL–V* (Dudley *et al.*, 2006). For the *S. sonnei* containing the IncK plasmid, the *bla*_CTXM-15_ gene was encoded along with the fluoroquinolone resistance enhancement gene *qnrS1* on an IS*26* transposon. The *S. sonnei* IncI1 plasmid (denoted pHUSEC2011-1-like) was almost identical ( ≥ 99 % DNA sequence identity) to the replication, conjugation and type IV pilus biosynthesis cluster *pilL–V* in pHUSEC2011-1. The pHUSEC2011-1-like plasmid was also independently acquired by six additional strains, although in these cases the *bla*_CTXM-15_ gene was not co-transferred (Fig. 2).

Lastly, nine *S. sonnei* strains isolated from cases occurring in June/July 2011 acquired an IncB-O plasmid, sharing homology with the MDR plasmid pHUSEC41-1 originally identified in an older O104 : H4 enteroaggregative Shiga toxin-producing *E. coli* (HUSEC41), which included a type IV pilus biosynthesis cluster *pilL–V* identical to that found on plasmid pSERB1 (Künne *et al.*, 2012). We further identified 11 *S. sonnei* strains harbouring a plasmid with homology to *Salmonella enterica* plasmid pSH146_65, additionally carrying a distinct type IV pilus cluster *pilL–V* (Han *et al.*, 2012).

## Discussion

Here, we have used WGS to define a population of *S. sonnei* causing disease in the South Asian land-locked nation of Bhutan. These strains belong to an expansion of lineage III, which we have named the Central Asia clade, which was introduced into Bhutan around 2006. To the best of our knowledge, this is the first time that WGS has been used on this scale to study a population of *S. sonnei* in South Asia, a region with a substantial burden of diarrhoeal disease. In contrast to recent observations from Vietnam, the sequenced *S. sonnei* in Bhutan demonstrated no evidence of a single clonal expansion. This is perhaps due to the short sampling time frame or other factors such as the lack of a definitive selective sweep event. However, the evolution history of Central Asia clade *S. sonnei* resembles the two initial sweep events observed in Vietnam [associated with fixation of a colicin plasmid and mutation(s) in *gyrA*] (Holt *et al.*, 2013), showing that the two geographically and phylogenically unrelated *S. sonnei* populations followed a similar evolutionary trajectory. After its introduction to Bhutan, our data predict that these *S. sonnei* had access to a substantial gene pool, as indicated by the sporadic acquisition of ESBL plasmids and type IV pili. The gains of ESBL plasmids, although infrequent, could potentially become the next selective sweep for the Bhutan's *S. sonnei* population as observed in Vietnam, given the selection path toward resistance is maintained. The characteristically thin type IV pilus has been shown to improve the efficiency and specificity of conjugation in liquid environments (Kim & Komano, 1997), and also facilitates adherence, interbacterial contact and twitching motility (Berry & Pelicic, 2015). Although this structure is known to be widespread in prokaryotes, its impact on the lifestyle of *Shigella* is not well described and requires further experimental investigation (Holt *et al.*, 2013; Rohmer *et al.*, 2014).

In addition, the Bhutanese *S. sonnei* population is characterized by the presence of numerous non-synonymous mutations, most of which are found in metabolic pathways. The genus *Shigella* is well known for its adaptive intracellular lifestyle, enabling its shedding of redundant or unnecessary functions during the course of evolution (Prosseda *et al.*, 2012). Our *in silico* analysis predicted that some mutations could affect metabolic activities, which could lead to degradation. In particular, the affected d-ribose utilization and formate fermentation appear to be redundant as energy production in *Shigella* predominantly occurs via the uptake and metabolism of pyruvate into acetate (Kentner *et al.*, 2014). Biosynthesis of histidine is also deemed inessential as the bacterium could readily transport it from the host's cytosol. However, the true impacts of the aforementioned mutations should be confirmed by functional analyses *in vitro* in future studies.

We were able to use WGS to identify the major events during the evolution of this lineage prior to, and after, its introduction into a new setting. Our data imply that the acquisition of a class 2 integron and a spA plasmid, together with the rapid accumulation of mutations associated with fluoroquinolone resistance, rendered the Bhutanese *S. sonnei* MDR by 2006. Furthermore, a fluoroquinolone-resistant subclade within the Central Asia clade was predicted to have arisen between 1999 and 2006. In addition, the fixation of a novel recombinant colicin E plasmid (pSSE3) may have offered this group of pathogens a competitive advantage over other enteric bacteria, similar to the colicin-associated selective sweep observed in *S. sonnei* in Vietnam (Holt *et al.*, 2013). These data suggest that whilst antimicrobial resistance is clearly important, it is not sufficient in itself for driving the spread of new clones and anticompetitive functions appear to be key themes in the spread of MDR *S. sonnei*.

Our data additionally suggests that international travel may have resulted in the dissemination of Central Asia clade *S. sonnei* beyond Bhutan and the remainder of the Indian subcontinent. *S. sonnei* isolates with similar PFGE patterns to the strains in this analysis have been recovered from cases of travel-associated shigellosis in Ireland (De Lappe *et al.*, 2015) and in a *Shigella* outbreak amongst ‘men who have sex with men’ in Canada (Gaudreau *et al.*, 2011). The *S. sonnei* isolated from both instances were found to be ciprofloxacin-resistant. Studies conducted in Japan have established a strong association between this pulsotype and travel to India, and increasing prevalence of nalidixic acid-resistant *S. sonnei* has been observed since 2001 (Uchimura *et al.*, 2001; Izumiya *et al.*, 2009). Shigellosis surveys in Iran conducted from 2008 to 2012 have repeatedly shown that this characteristic Asian pulsotype is prevalent and endemic, and that these strains also possess a similar class 2 integron (*dfrA1*, *sat1*), but exhibit variability in their ciprofloxacin susceptibility phenotype (Tajbakhsh *et al.*, 2012; Eftekhari *et al.*, 2013; Alizadeh-Hesar *et al.*, 2015). The first case of fluoroquinolone-resistant *S. sonnei* was identified in Japan in 1993 (Horiuchi *et al.*, 1993). Since then, the incidence of ciprofloxacin-resistant *Shigella* in Asia and Africa has risen dramatically, from 3.9 % in 2001–2003 to 29.1 % in 2007–2009 (Gu *et al.*, 2012). The potential emergence and dissemination of fluoroquinolone-resistant *S. sonnei* poses a substantial threat to public health, including low shigellosis burden areas in developed nations, as again substantiated by the recent fluoroquinolone-resistant *S. sonnei* outbreak in the USA (Bowen *et al.*, 2015). Taken together, these data strongly suggest that the Central Asia clade of *S. sonnei* is widely present across South Asia and beyond, and may represent an emerging threat to public health.
